# Optimising patient engagement for assessing total joint arthroplasty outcomes – a randomised controlled trial

**DOI:** 10.1186/s13018-025-05493-w

**Published:** 2025-01-23

**Authors:** Wyatt Tyndall, Nebojsa Kuljic, Michael Thatcher, Michaela Nickol, Johannes M. van der Merwe

**Affiliations:** Department of Orthopaedic Surgery, College of Medicine, University of Saskatchewan, Saskatoon, SK Canada

**Keywords:** Patient reported outcome measures, Joint arthroplasty, PROMS, Response rates, Mailed questionnaires, Telephone calls

## Abstract

**Introduction:**

Patient satisfaction is a critical outcome in total joint arthroplasty (TJA), yet assessing it effectively remains a challenge due to limitations in patient-reported outcome measures (PROMS). While these measures are commonly gathered in clinical settings, additional contact through mail or phone is often needed, and low response rates can affect the validity and reliability of collected data. To improve response rates, this study evaluated various methods of incentivizing patient participation in a randomized trial format, focusing on postal questionnaires.

**Patients and methods:**

The study investigated three methods to improve response rates: including a gift card with the questionnaire, promising a gift card upon questionnaire completion, and offering no incentive. It also examined whether different monetary values and the inclusion of the surgeon’s name on materials impacted response rates. We tried to determine factors that could improve follow up telephone response rates in the group of patients that failed to return their questionnaires.

**Results:**

Higher response rates were observed with monetary incentives (*P* = 0.056), larger amounts of money offered (*P* = 0.3839) for filling out the questionnaire, and if the surgeon’s details were on the cover letter or questionnaire (*P* = 0.632). There was no correlation between age and sex and participation. We did find a statistically significant difference in total participation and poorer total knee arthroplasty outcomes scores (*P* < 0.001).

**Conclusion:**

Our study supports findings from prior research indicating that monetary incentives and personalized materials can improve response rates, although in this cohort, results were modest. Follow-up calls further boosted response rates, suggesting that multi-modal engagement may be beneficial. Although the response improvements were limited and lacked statistical significance, the study highlights the importance of refining strategies to ensure reliable PROMS data, which is vital for understanding patient outcomes in TJA. Future studies might consider demographic factors and other outreach methods to enhance PROMs data collection.

## Introduction

Patient satisfaction is becoming increasingly important in total joint arthroplasty. Currently there isn’t a perfect tool to assess patient satisfaction and surgeons rely on patient reported outcome measures (PROMS) to determine patient satisfaction. PROMS are now routinely collected in the office in a prospective manner but periodically patients have to be contacted via postal mail or telephone to obtain these questionnaires. The response rate to questionnaires is crucial because low response rate reduces the effective sample size and can introduce bias [1–3]. Ensuring a high response rate to initial mailings has the additional benefit of reduced costs associated with re-mailing or telephone interviews [2].

The strategies to improve response rates can be summarised into five main categories. The first is the cover letter (to improve the appeal of the letter or personalization); secondly to offer an incentive (either monetary or non-monetary e.g. pen, or enter into a draw); thirdly patient contact (pre notification and follow up); Fourth mailing (include a return envelope, include postage etc.) and lastly the questionnaire (length, format, colour).

In a recent Cochrane review [1] which included 758 studies, it was found that contacting people prior to be sent a questionnaire improved response rate. Other methods to improve response rates included if the questionnaire was sent from a university, or if patients received a small incentive (monetary or non-monetary incentive e.g., pen). They also concluded that response rates may be higher if a postal questionnaire was used instead of an electronic questionnaire. In addition, making the letter or questionnaire more personal and shorter improved response rates. Even though this review article was well conducted it did include a variety of participants (patients, doctors, university students, professors, marketing managers, accountants and grocery store managers) which makes the application to orthopaedic patients difficult. In addition, the studies included in this review was from a wide variety of specialties (psychology, medicine, business etc.) and was conducted in a variety of countries, making their generalisability questionable.

Currently, evidence is lacking in the orthopaedic literature which methods work best for collecting PROMS in arthroplasty patients. We therefore conducted a randomised trial aiming to determine which methods would improve response rates using postal mail questionnaires.

Our primary objective was to determine which of three methods would lead to a higher response rate: including a gift card, without an obligation to fill out the questionnaire, in the mailed-out envelope; or the promise of a gift card once the completed envelope is mailed back or lastly no incentive offered with filling out the questionnaire. Our secondary objectives were to determine if return rates of envelopes were influenced by monetary value. We promised different monetary values ($5, $10, $15) to see if larger monetary values would improve response rates. In addition, we tried to calculate if the surgeon’s details on the cover sheet and questionnaire, or lack of it, influenced patients’ willingness to participate. Lastly, we tried to determine factors that could improve telephone response rates in the group of patients that failed to return their questionnaires.

## Patients and methods

**Fig. 1:**
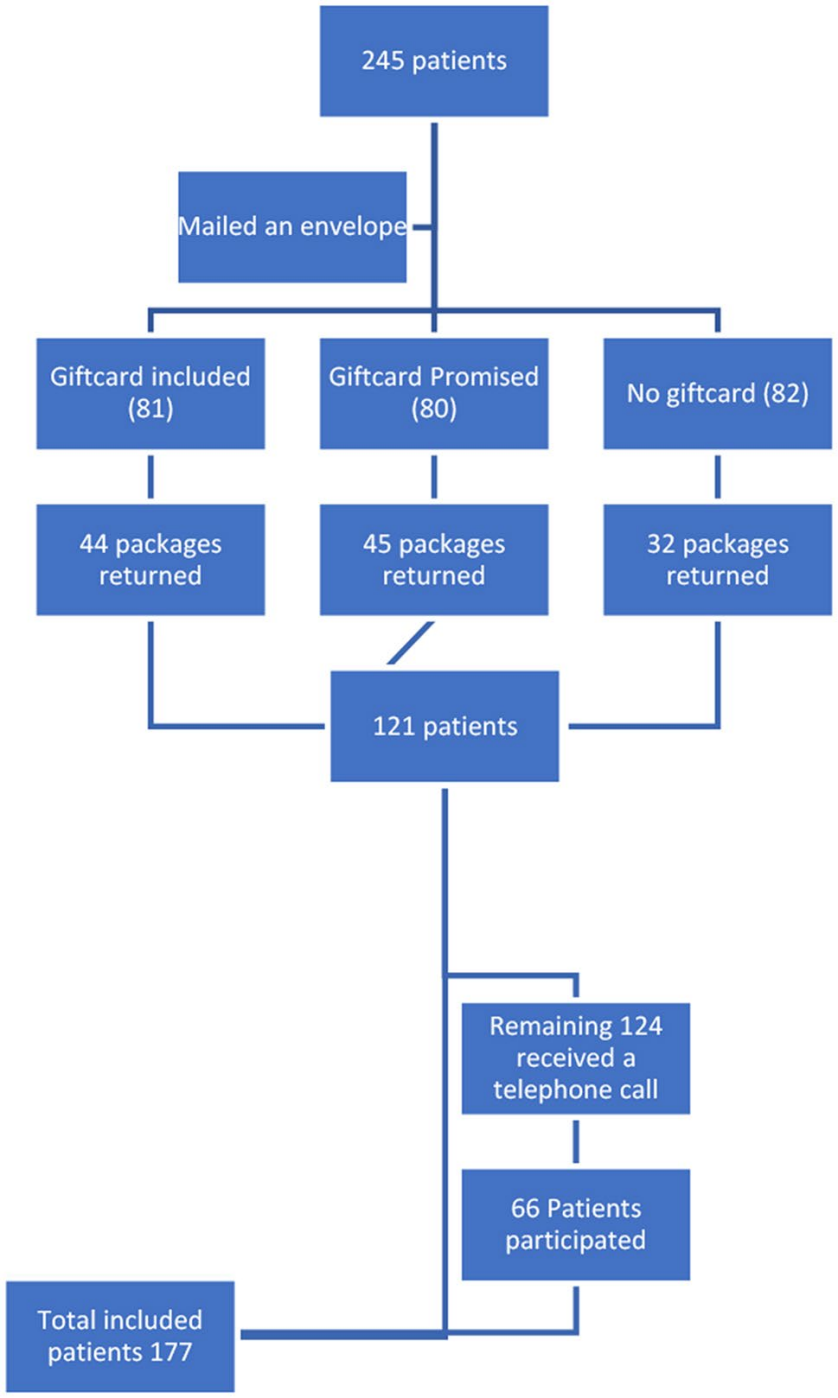
Flow diagram of patient participation in part 1

The study adhered to stringent ethical guidelines, obtaining approval from the Bio-REB 8834. The methodology employed a systematic approach to ensure ethical standards were maintained throughout the study. The study was conducted in three parts, each of which served as an extension of a separate prior study. In each part, we investigated different aspects of patient communication. These add-on studies allowed for a more comprehensive examination of communication dynamics between the surgeon and patients (See Figure 1).

**Fig. 2:**
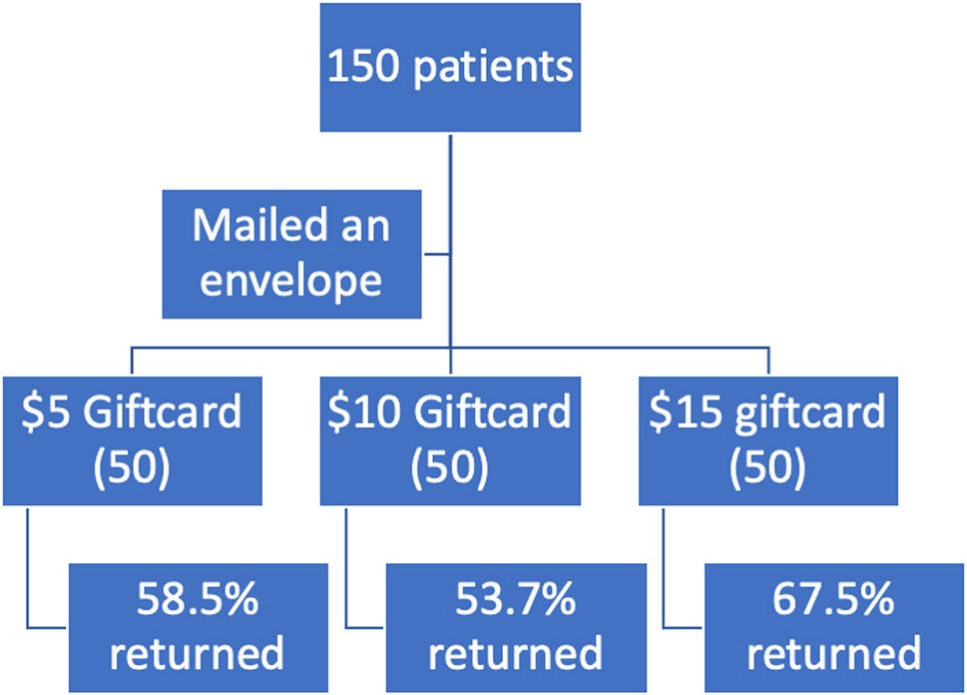
Flow diagram of patient participation in part 2

In the first part we mailed out questionnaire packages (KOOS JR Knee Survey, Oxford Knee Score Survey, and the KUJALA Questionnaire) to patients that received total knee arthroplasty surgery between January and December 2020. To encourage participation, patients were randomly assigned to one of three incentive groups. The first group received a $10 gift card enclosed with the questionnaire package, with no obligation to complete the survey. In the second group, patients were promised a $10 gift card upon returning the completed questionnaire. The third group received no financial incentive. This randomized allocation aimed to assess the impact of different incentive structures on patient response rates and data quality. Each questionnaire package included a pre-addressed return envelope and postage stamp, simplifying the process for patients to return their completed surveys. All packages were dispatched simultaneously to ensure consistency and fairness in participant recruitment. An 8-week period was allocated for patients to return the questionnaires by mail. Following the mail-in phase, patients who had not returned their questionnaires were contacted via telephone to encourage participation. The telephone contact protocol involved making two attempts to reach each patient. During the telephone contact phase, additional information was documented e.g., patient demographics such as sex and age, as well as the time of day when the call was made and whether the patient requested a callback (See Figure 2).

**Fig. 3:**
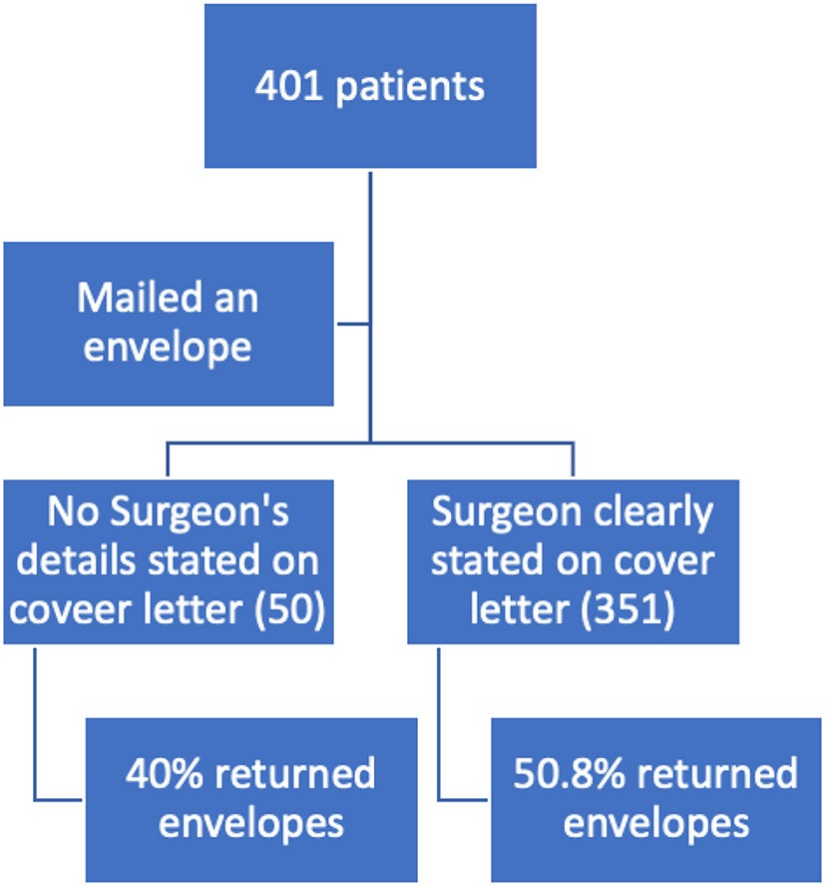
Flow diagram of patient participation in part three

In the second part we mailed out questionnaires (Oxford Hip Score and Quality of Life Score) to 150 patients to patients that underwent total hip arthroplasties between January 2022 and January 2024. The first 50 patients were promised a $5 gift card. The second and third group were promised a $10 and $15 gift card respectively. The mail packages were appropriately randomised. We conducted the second part of the study using the same methodology as the previous part, ensuring consistency in the approach and allowing for direct comparison of the results. We then calculated the response rate of returned envelopes within an 8-week period. Any envelopes not returned during that period were excluded from the analysis (See Figure 3).

In the third part of the study, we mailed out 401 envelopes containing questionnaires (KOOS JR Knee Survey, Oxford Knee Score Survey) to 150 patients to patients that underwent total hip arthroplasties between January 2022 and January 2024. to patients that underwent total knee arthroplasty surgery in 2017–2018. The first 50 envelopes did not contain the surgeon’s name on either the questionnaire or information sheet explaining the purpose of the study. The following 351 envelopes did contain the operating surgeon’s name. We again conducted the third part of the study using the same methodology as the previous parts, ensuring consistency in the approach and allowing for direct comparison of the results. We calculated the response rate of returned envelopes within an 8-week period. Any envelopes not returned during that period were excluded from the analysis.

### Statistics

#### Group comparison

The three groups, included in this study, were determined by whether a gift card was included in the mailing package. Group 1 received a gift card included, Group 2 received a gift card upon completion, and Group 3 did not receive a gift card. Both groups demographic data as well as methods of questionnaire completion were analysed to ensure that cases and controls are comparable. After that, the questionnaire scores were compared to determine the difference between groups. Fisher’s exact test was employed for comparison.

Fisher’s exact test is a statistical test used to determine if there are nonrandom associations between two categorical variables in a relatively small sample size. It calculates the probability of observing a particular arrangement of data assuming that the null hypothesis (no association) is true.

#### Correlation

Correlation is used to measure the strength and direction of the relationship between two variables, helping to understand how changes in one variable relate to changes in another. Correlation was assessed both between the group designation and the dataset, using either Cramer’s V or Kendall’s Tau.

Cramer’s V is a statistical measure used to assess the strength of association between categorical variables. It ranges from 0 to 1, with 0 indicating no association and 1 indicating a perfect association.

Kendall’s Tau is a statistic used to measure the association or correlation between two sets of ranked data. Kendall’s Tau ranges from − 1 to 1, where 1 indicates perfect agreement in rankings, -1 indicates perfect disagreement, and 0 indicates no association. It is robust to outliers and does not assume any specific distribution of the data.

We used a chi-square test of independence to determine if there is a significant difference in response rates among the three groups. Comparing the three groups independently to each other we used Fisher’s exact test.

## Results

### Part one

**Fig. 4:**
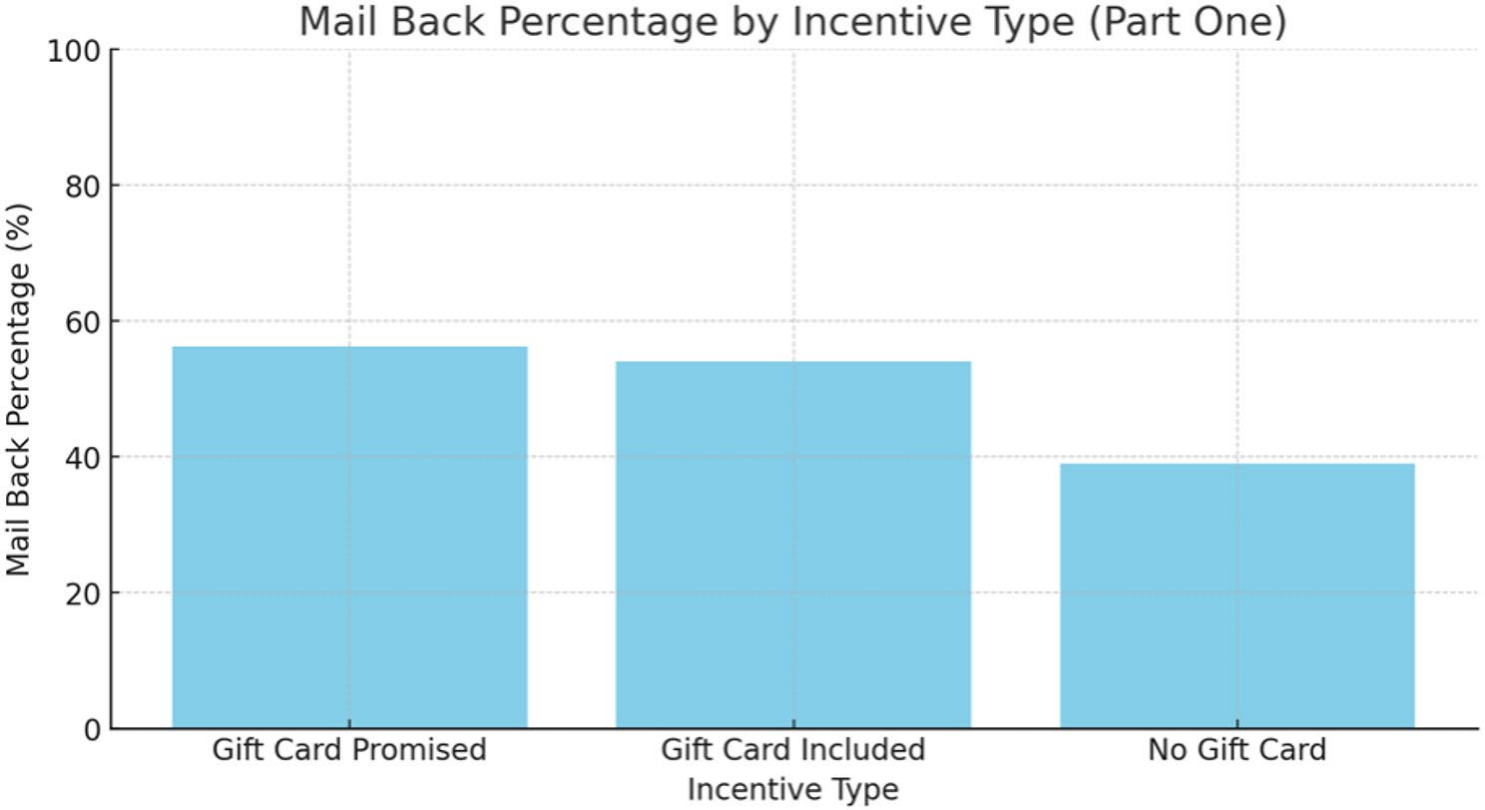
Mail back percentages of gift cards. This was not statistically significant (*p* = 0.056)

There were 245 patients in total that received a mailed-out package. Out of the 245 patients 121 patients returned the packages (49.38%). Group 2 (gift card promised) had the highest mail back percentage of 56.2% (45/80) followed by group 1 (gift card included), 54% (44/81) and group 3 (no gift card), 39% (32/82) (See Tables 1 and 2 and Fig. 4). This was not statistically significant (*P* = 0.056). Comparing the completeness of the returned packages between the three groups there was no statistically significant difference (*p* = 0.762).

**Table 1:** Cross table between the 3 groups. No significant difference between the groups (*P* = 0.056)

	Group	Package Mailed Back?		Total
		No	Yes	
Assignment	1	37	44	81
	2	35	45	80
	3	50	32	82
Total		122	121	243

**Table 2:** Cross table between the three groups comparing the completeness of the returned mail packages. No significant differences between the groups (*P* = 0.762)

	Groups	Package Completed Satisfactorily?		Total
		No	Yes	
Assignment	1	20	54	74
	2	17	61	78
	3	19	54	73
Total		56	169	225

The remaining 124 patients received a telephone call from which only 66 agreed to participate (53.22%). The majority of the telephone calls occurred in the morning (70%). There was no correlation between sex and time of day the questionnaires were filled out via a telephone call. (*p* = 1.000) (See Table 3). Fifty-nine patients participated in the initial telephone call (47.5%) while 13 patients (10.5%) requested a call back at another time. Only 53.8% of the patients requesting a call back decided to finally participate. There was no correlation between participation by mail or phone calls and age (Pearson *R* = 0.015). There was no correlation between sex and participation by mail or phone (Cramer’s V = 0.074). There was no correlation between age and sex and the three study groups (Cramer’s V – 0.080, Kendall’s Tau = -0,094).

**Table 3:** Cross table between male/female participation via telephone calls and time of day. No significant difference (*P* = 1.00)

Time of day participant Crosstabulation				
Count				
		Participants		Total
		Male	Female	
Time of day	Am	5	7	12
	Pm	5	9	14
Total		10	16	26

There was a higher correlation between a higher KOOS JR score and total participation (Kendall’s Tau = 0,249; *p* < 0,001).

### Part two

**Fig. 5:**
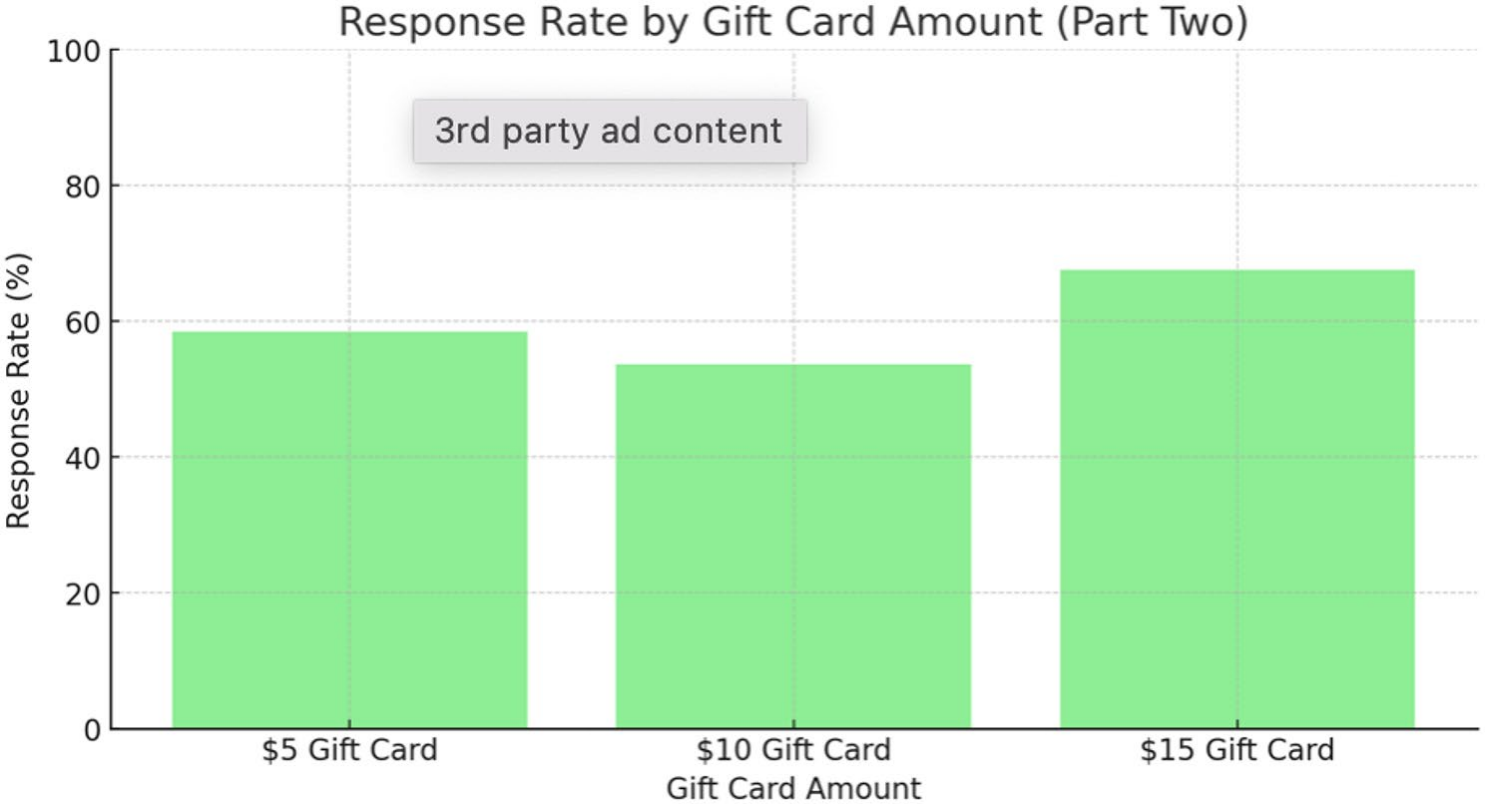
Response rate by gift card amount (*p* = 0.3839)

Out of the 150 mailed out packages only 90 patients returned their questionnaires (60% response rate). The response rate between the three groups was 58.5% in group A ($5 gift card); 53.7% in group B ($10 gift card) and 67.5% in group C ($15 gift card) (*p* = 0.3839) (See Fig. 5). When we compared the three groups independently to each other we also did not detect a statistical significance. Group A compared to Group B (*p* = 0.6602; odds ratio: 1.22); Group B vs. C (*p* = 0.1904; OR 0.55); Group A vs. C (*p* = 0.3921; OR 0.68).

### Part three

**Fig. 6:**
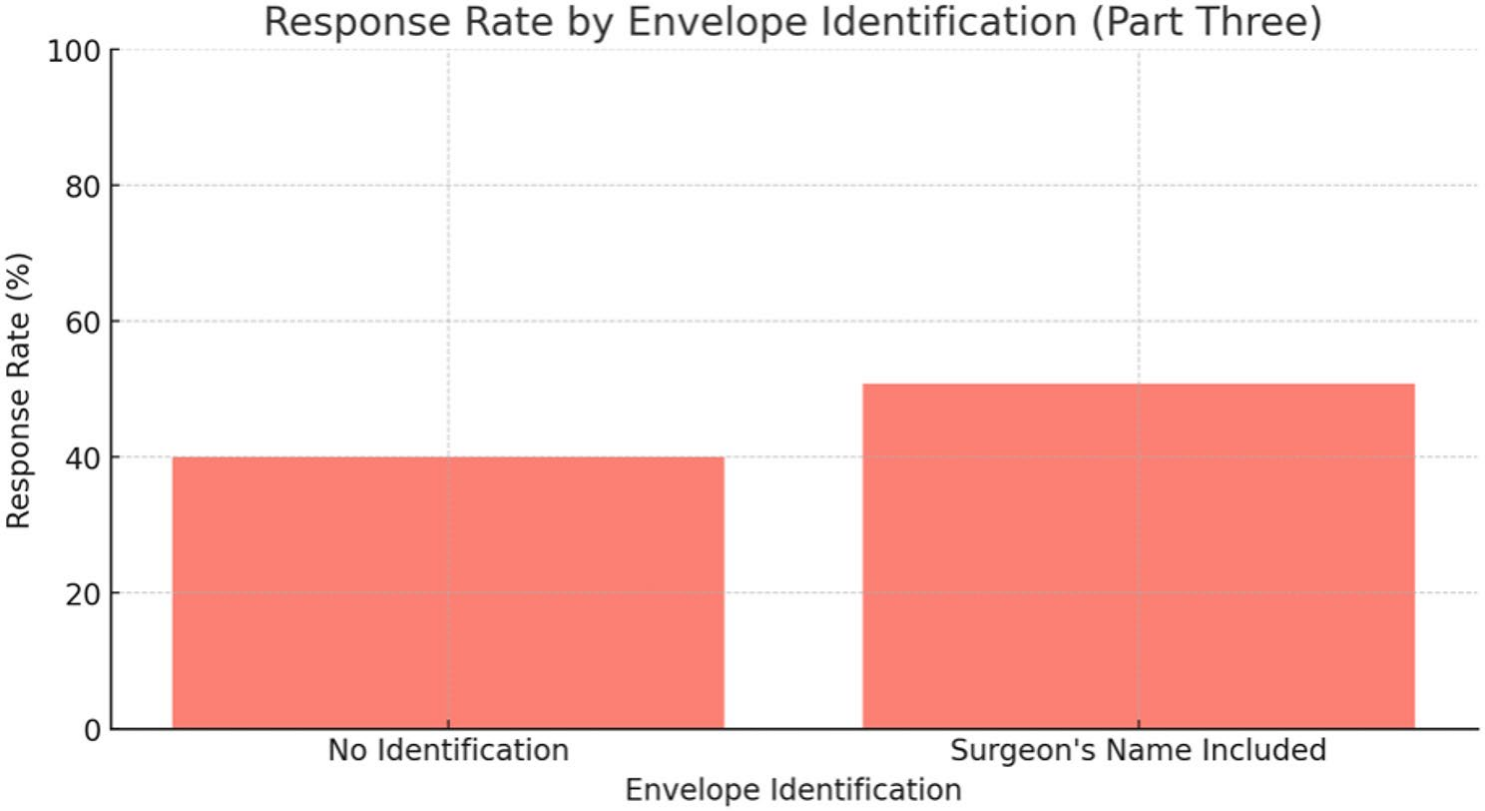
Response rate by envelope identification (*p* = 0.632)

There were 401 patients that received a mailed-out questionnaire. Only 173 returned their envelopes (43.1%). Patients were more inclined to return the envelopes that contained the surgeon’s name compared to generic envelopes without any identification. In the group without identification 40% (20/50 envelopes) were returned, compared to 50.8% (153/351 envelopes) returned in the group that clearly identified their surgeon as the investigator (*p* = 0.632) (See Fig. 6).

## Discussion

In this randomised trial we determined that patients were more inclined to return their questionnaires if there was an incentive promised upon receipt of the completed questionnaire. Higher response rates were also observed with larger amounts of money offered for filling out the questionnaire and if the surgeon’s details were on the cover letter, envelope or questionnaire. None of these findings were however statistically significant. There was no correlation between age and sex and participation. We did find a statistically significant difference in total participation and poorer total knee arthroplasty outcomes scores. With follow up telephone interviews most interviews occurred in the morning.

Multiple studies confirmed our results that monetary values lead to higher response rates [4, 5, 7, 8]. One systematic review including 258 315 patients found that the odds ratio more than doubled (2.02) with using a monetary incentive [5]. They also found that the odds ratio nearly doubled (1.71) if a monetary incentive was included non-conditional on response. In another systematic review with 109 648 patients, they concluded that monetary incentives improved response rates. Their findings were similar to ours where larger monetary incentives lead to better response rates [7]. Interestingly, money was considered to be the best incentive compared to other modalities e.g., coupons, pens etc [7].

Providing an incentive in advance not contingent on returning the questionnaire has been shown in the literature to increase response rates [23, 24]. It is based on the premise of building a trusting relationship between the researcher and the patient which then subsequently could lead to an increased response rate [1, 22]. We did not find a statistically significant difference utilizing the unconditional incentive. Koloski et al. [22] had similar findings with 450 patients. In contrast Wiant et al. [23] and Leung et al. [24] did find a statistically significant increase in response rates using the unconditional incentive.

Multiple studies confirmed our results that response rates improved with university or surgeon’s details on the questionnaires [1, 8–11]. However, one study involving 500 participants did not find an increase in response rates when the questionnaires were sent in a university printed envelope [12]. Two studies evaluated the effect of a detailed cover letter on response rates and found no statistical difference [14, 15]. In addition, multiple studies did not find a difference in response rates if there was an appeal to fill in the questionnaires [ 12,13].

We increased our response rates with a follow up telephone call for the patients that didn’t return their questionnaires. We were able to increase our response rate from 49.38 to 76.32%. This was different from prior studies which did not see a difference [1, 16, 17]. Garcia et al. [18] determined that telephone interviews only, had the best response rates followed by mailed questionnaires and follow-up telephone interviews; or mailed questionnaires and follow-up reminder letters. However, participants rated mailed questionnaires as their preference. This was in stark contrast with other studies which found that telephone reminders were better than an interview [16, 17]. One study showed resending a questionnaire demonstrated a higher response rate than contacting people via phone [17].

We found the best patient participation in the morning with the follow up telephone interviews. Even though the orthopaedic literature is lacking in timing of day and participation, there are studies that confirm higher participation with surveys sent earlier in the week and in the morning hours [25, 26]. One study found increased response rates if e-mail invitations were sent on a Tuesday morning [25]. Another study found higher response rates with internet surveys on Wednesday mornings [26].

Interestingly we noticed an increase in response rates with poorer outcomes on PROMS. This was in contrast with Perneger et al. [20] and Mazor et al. [21] who demonstrated larger response rates with patients that were satisfied with their surgery. They also found an increase in patient responses in the group of patients least satisfied. Perneger et al. [20] also noticed an increase in patient responses in the group of patients least satisfied. This might explain why we saw an increase in response rates with poorer KOOS JR scores.

### Limitations

Our study does have several limitations. Firstly, we didn’t control for demographic differences. Variables like geographic location, socio-economic status, education level and cultural background could have influenced the results. We also only used a single method of data collection in part two and three which could lead to underrepresentation in patients with limited literacy, language barriers and mobility issues. In addition, there may be limited statistical power to detect small differences in the three-part analysis.

### Authors current practise

Currently, patients complete Patient-Reported Outcome Measures (PROMs) during each clinical visit. For patients undergoing total knee arthroplasty (TKA), the Knee injury and Osteoarthritis Outcome Score for Joint Replacement (KOOS JR) and the Oxford Knee Score are utilized, while for total hip arthroplasty (THA), the Oxford Hip Score and Quality of Life Score are administered. For patients whose follow-up predates the implementation of PROMs at our practice, we email the relevant surveys. In cases where surveys are mailed, the package includes a return envelope with prepaid postage, clear documentation identifying the source as the surgeon or affiliated university, and an incentive in the form of a gift card upon completion of the questionnaire.

## Conclusion

This study aimed to evaluate the most effective method of reaching patients for assessing total joint arthroplasty outcomes. While no statistically significant difference was observed among the incentive groups in terms of mail-back response rates, the study sheds light on the importance of strategies like including monetary incentives, ensuring the cover letter or envelope clearly represents the surgeons or university’s details and utilising telephone follow-ups. Moreover, the findings highlight the relevance of patient-reported outcome measures (PROMs), emphasising their pivotal role in understanding patient satisfaction following orthopaedic procedures. Further research could delve into refining methods for maximising patient engagement and optimising data collection processes in orthopaedic practice.

## Data Availability

No datasets were generated or analysed during the current study.
